# Sperm Cryopreservation in American Flamingo (*Phoenicopterus Ruber*): Influence of Cryoprotectants and Seminal Plasma Removal

**DOI:** 10.3390/ani11010203

**Published:** 2021-01-15

**Authors:** María Gemma Millán de la Blanca, Eva Martínez-Nevado, Cristina Castaño, Juncal García, Berenice Bernal, Adolfo Toledano-Díaz, Milagros Cristina Esteso, Paula Bóveda, Lucía Martínez-Fresneda, Antonio López-Sebastián, Julián Santiago-Moreno

**Affiliations:** 1 Department of Animal Reproduction, Instituto Nacional de Investigación y Tecnología Agraria y Alimentaria (INIA), 28040 Madrid, Spain; gemma.millan@inia.es (M.G.M.d.l.B.); cristina.castano@inia.es (C.C.); berenice.bernal@inia.es (B.B.); toledano@inia.es (A.T.-D.); esteso.milagros@inia.es (M.C.E.); boveda.paula@inia.es (P.B.); lumf4@msn.com (L.M.-F.); alopezs@inia.es (A.L.-S.); 2 Zoo-Aquarium Madrid, 28011 Madrid, Spain; emartinez@grpr.com (E.M.-N.); jgarcia@grpr.com (J.G.)

**Keywords:** cryopreservation, American flamingo, spermatozoa, freezing/thawing, cryoprotectant, seminal plasma, morphometry

## Abstract

**Simple Summary:**

The Phoenicopteridae family is made up of six species of which 50% are in near-threatened status and 17% are in vulnerable status because their population is decreasing. The American flamingo is a useful model for development of successful semen cryopreservation procedures to be applied to threatened related species from the family Phoenicopteridae, and to permit genetic material banking. The current study sought to develop effective sperm cryopreservation protocols through examining the influences of two permeating cryoprotectants and the seminal plasma removal. In addition, morphometric and functional sperm characteristics were studied for the first time in this species. Head morphometric data provide relevant information in future studies about sperm cryobiology in these species because the head size is related with the ability of sperm to survive the freeze/thawed process. There was no apparent effect of cryoprotectants (DMA (dimethylacetamide) 6% and Me_2_SO (dimethylsulphoxide) 8%) on frozen–thawed flamingo sperm variables. The removal of the seminal plasma provided lower sperm quality after thawing than samples containing seminal plasma. This research demonstrated that there were no differences between Me_2_SO and DMA to successful freezing sperm of flamingos, but we recommend the use of Me_2_SO because the cell toxic effect of DMA.

**Abstract:**

The American flamingo is a useful model for the development of successful semen cryopreservation procedures to be applied to threatened related species from the family Phoenicopteridae, and to permit genetic material banking. Current study sought to develop effective sperm cryopreservation protocols through examining the influences of two permeating cryoprotectants and the seminal plasma removal. During two consecutive years (April), semen samples were collected and frozen from American flamingos. In the first year, the effect of two permeating cryoprotectants, DMA (dimethylacetamide) (6%) or Me_2_SO (dimethylsulphoxide) (8%), on frozen–thawed sperm variables were compared in 21 males. No differences were seen between DMA and Me_2_SO for sperm motility, sperm viability, and DNA fragmentation after thawing. In the second year, the role of seminal plasma on sperm cryoresistance was investigated in 31 flamingos. Sperm samples were cryopreserved with and without seminal plasma, using Me_2_SO (8%) as a cryoprotectant. The results showed that samples with seminal plasma had higher values than samples without seminal plasma for the following sperm variables: Straight line velocity (22.40 µm/s vs. 16.64 µm/s), wobble (75.83% vs. 69.40%), (*p* < 0.05), linearity (62.73% vs. 52.01%) and straightness (82.38% vs. 73.79%) (*p* < 0.01); but acrosome integrity was lower (55.56% vs. 66.88%) (*p* < 0.05). The cryoresistance ratio (CR) was greater in samples frozen with seminal plasma than without seminal plasma for CR-progressive motility (138.72 vs. 54.59), CR-curvilinear velocity (105.98 vs. 89.32), CR-straight line velocity (152.77 vs. 112.58), CR-average path velocity (122.48 vs. 98.12), CR-wobble (111.75 vs. 102.04) (*p* < 0.05), CR-linearity (139.41 vs. 113.18), and CR-straightness (124.02 vs. 109.97) (*p* < 0.01). This research demonstrated that there were not differences between Me_2_SO and DMA to successful freezing sperm of flamingos; seminal plasma removal did not provide a benefit for sperm cryopreservation.

## 1. Introduction

The Phoenicopteridae family is made up of six species which the 50% is in near threatened status and the 17% is in vulnerable status because their population is decreasing. The principal threats are the collection of eggs as food, mining activities, unfavorable water-levels, hyper-salinity of water, erosion of nest-sites, human disturbance, pollution and hunting food [1]. Currently, the Chilean flamingo (*Phoenicopterus chilensis*) is near threatened and Andean flamingo (*Phoenicoparrus andino*) is included in the IUCN red list of threatened species as vulnerable. [1]. In this context, reproductive biotechnologies, particularly cryopreservation of gametes, and the development of Genetic Resource Banks provide us with an indefinite gene resource of these species. The American flamingo (*Phoenicopterus ruber*) is a good animal model for the study of the sperm cryopreservation of flamingo species because its phylogenetic relationship with threatened species and its least concern status [1].

The storage of sperm in genetic resource banks allows maximizing the genetic diversity, and the sustainability of captive populations [2]. Some aspects to take into account for semen cryopreservation are the cooling rate [3], the semen packaging type (e.g., straws or pellets) [4], the presence or absence of seminal plasma [5], and the cryoprotectant agent used [6]. To the best of our knowledge, there are no studies on the semen cryopreservation of flamingo species.

Glycerol is generally thought to be the most effective cryoprotective agent in avian and mammalian sperm cells against cold shock and it is the least toxic toward them [7]. Glycerol is the most effective cryoprotectant in low fertility lines of poultry [7]. Although the use of glycerol is associated in poultry with high sperm motility and viability after thawing, its use has been shown to have contraceptive effects in birds, and therefore glycerol must be eliminated from frozen–thawed semen before insemination [6,8,9]. Some alternatives to consider are the Me_2_SO (dimethyl sulphoxide) [10] or DMA (dimethylacetamide) [3,4]. The Me_2_SO is a permeable cryoprotectant that increases the fluidity lipid membrane’s hydrophobic core (but a high concentration can disintegrate the lipid bilayer); enhances the permeability of hydrophilic molecules [11]; and decreases the ice crystallization [12]. The use of 8% Me_2_SO improved the frozen–thawed sperm quality and the fertility (73% fertility) in Indian red jungle fowl compared with other concentrations [10], and has been successfully used in crane sperm, obtaining a higher post-thaw survival and capacity to bind to inner perivitelline membrane than with DMA [13]. On the other hand, DMA has provided high levels of fertility after artificial insemination with frozen–thawed fowl (85–93% fertility) and sandhill crane (42–48% fertility) sperm [4,14,15]. Its use at 6% in a 2-step freezing method allows a good preservation of acrosome and membrane integrity, and 41% fertility in chicken sperm [3].

Seminal plasma is a key biological fluid that modulates sperm function in all animal species. In mammals, the seminal plasma may have some components that protect of cryoinjury and improve the sperm resistance to cold-shock damage [16,17,18], but in some species (e.g., goat) the seminal plasma removal is routine in sperm cryopreservation protocols, because contains components that reduces the chances of efficient sperm preservation [19]. Some of these components include proteins, ions, free amino acids, lipids, carbohydrates, polyamines, steroid hormones, and prostaglandins [5,20,21,22,23,24]. The role of seminal plasma on bird semen in vitro storage remains largely a matter of speculation as both inhibitory and stimulating effects have been found [20]. The intense sperm metabolism and the high proteolytic activities of seminal enzymes imply very rapid dilution of bird semen in buffered diluents to avoid the rapid degradation of sperm in vitro [20]. Previous reports showed contrasted effects of seminal plasma fractions on sperm cryopreserved or incubated, showing a global deleterious effect on chickens and turkey sperm [25,26,27]. In chickens the fertilization rate was lowest in older animals when the semen was stored with seminal plasma, and in turkey the sperm membrane integrity, sperm motility, energy status, and fertility were lower in semen samples stored in presence of seminal plasma [25,27]. Whereas the presence of seminal plasma did not seem to affect the rooster sperm survival after freezing and thawing, the DNA was less damaged without plasma [5].

Considering all this information, the aim of this study was to develop a suitable method for cryopreservation of flamingo’s semen by comparing the effect of two cryoprotectant agents, Me_2_SO and DMA, and evaluating the effect of seminal plasma removal on sperm cryoresistance. Morphometric and functional sperm characteristics were studied for the first time in this species.

## 2. Materials and Methods

### 2.1. Animals

American flamingo (*Phoenicopterus ruber*) males were housed in an outdoor exhibition enclosure at Madrid Zoo-Aquarium (Madrid, Spain) in natural condition of photoperiod and temperature. For the first experiment, 21 flamingos were sampled and for the second experiment 31 males were sampled. All animals of the first experiment also participated in the second experiment. The management of the bird and the semen collection was done during the annual routine health check and was applied the Spanish Policy for Animal Protection (RD53/2013), which conforms to European Union Directive 2010/63/UE regarding the protection of animals used in scientific experiments. Animals were handled according to procedures approved by the INIA Ethics Committee (Reference number ORCEEA 2016/001). In addition, the internal animal welfare committee of Madrid Zoo-Aquarium, based on the EAZA (European Association of Zoos and Aquariums) Code of Ethics, evaluated and approved all procedures of this research.

### 2.2. Semen Collection

The semen samples were collected for two consecutive reproductive seasons, once a year in April. All samples for each experiment were collected in only one day. The first year the samples were used to investigate the effect of two cryoprotectants, Me_2_SO and DMA. The second year the samples were used to evaluate the effect of seminal plasma removal on sperm cryoresistance. All birds were subjected to the massage technique of Burrows and Quinn, adapted to this species [28]. The collection of semen required some research staff. One individual immobilized the body, the head and the neck, other held the legs in the floor, and other stimulated the bird and collected the sample. The stimulation was done massaging the abdomen with the left hand, the back with the right hand and finally the copulatory organ protruded by mild stimulation and then gripping its base with the thumb and index fingers of the right hand. Semen was recovered by capillarity using a microhematocrit tube (Brand^®^ GMBH + Co KG, Wertheim, Germany), and the volume was evaluated measuring the length of the semen column in the microcapillary tube with a plastic ruler (accuracy ± 1 mm) and calculating the equivalent in volume units (μL). The microcapillary was emptied into a 1.5 mL Eppendorf microcentrifuge tube (Eppendorf Ibérica SLU, Madrid, Spain), and the semen sample was diluted 1:1 (*v:v*) at ambient temperature with Lake-Ravie medium composed of sodium-L-glutamate (1.92 g), glucose (0.8 g), magnesium acetate 4H2O (0.08 g), potassium acetate (0.5 g), polyvinylpyrrolidone (PVP, relative molecular mass = 10,000; 0.3 g), and H2O (100 mL) (final pH 7.08, final osmolarity 343 mOsm/kg). The diluted semen samples were immediately refrigerated at 5 °C (cooling rate: 0.2 °C/min) and transported to the laboratory. 

### 2.3. Semen Freezing

Experiment 1: All the semen samples were divided in two aliquots and diluted with Lake-Ravie medium to a concentration of 800 million spermatozoa/mL. The aliquots were incubated 5 °C for 1 h. Afterwards, the cryoprotectant was added to the respective aliquots to a final concentration of 6% DMA and 8% Me_2_SO. The aliquots kept for equilibration at 5 °C for 10 min. After equilibration, these samples were loaded into 0.25 mL French straws (Minitub, Landshut, Germany). These straws were then frozen by a two-step cooling method (from 5 °C to −35 at 7 °C/min, and from −35 °C to −140 °C at 60 °C/min) [3]. First, the straws were frozen by placing them in nitrogen vapor 17 cm above the surface of a liquid nitrogen bath for 4 min, and after were placed in nitrogen vapor 1 cm above the surface of a nitrogen bath for 2 min (box’s volume 4.24 L). These samples were then plunged into the liquid nitrogen.

Experiment 2: All the semen samples were divided into two aliquots and diluted with Lake-Ravie medium to a concentration of 800 million spermatozoa/mL. The samples were centrifuged to 700 *g* 10 min. The pellet of one aliquot was resuspended with its own seminal plasma. In the other one, the seminal plasma was removed and the pellet resuspended with the same volume of Lake Ravie medium. The aliquots were incubated 5 °C for 1 h. Afterwards, the Me_2_SO was added resulting in final concentrations of 8 vol%. We decided to use this cryoprotectant agent because in the first experiment there were not significates differences among both cryoprotectants, and the DMA has an effect very toxic in rooster sperm at high concentration [29]. The samples were equilibrated 10 min with the cryoprotectant and frozen as described in the experiment 1. 

### 2.4. Semen Thawing

The straws were thawed in a bath at 5 °C for 3 min and they were kept at this temperature in a refrigerated display case until sperm variables were analyzed [2].

### 2.5. Assessment of Semen Variables

#### 2.5.1. Sperm Motility 

First, all semen was examined using a phase contrast microscope to confirm the presence of spermatozoa and the subjective motility (percentage of motile spermatozoa and score of 0 to 5 depend of quality of movement) [3]. Afterwards, sperm concentrations and motility were assessed using a computer-aided sperm analysis (CASA) system coupled to a Nikon Eclipse model 50i phase contrast microscope (Nikon Instruments Europe B.V., Izasa S.A., Barcelona, Spain; negative contrast mode) and a Sperm Class Analyzer v.4.0. Software (Microptic S.L., Barcelona, Spain). The samples were diluted in Lake-Ravie medium and loaded onto warmed (37 °C) 20 μm Leja^®^ 8-chamber slides (Leja Products B.V., Nieuw-Vennep, The Netherlands). The program determined the percentage of motile spermatozoa, the kind and characteristics of sperm movement—curvilinear velocity (VCL), straight-line velocity (VSL), average path velocity (VAP), amplitude of lateral head displacement (ALH), and beat-cross frequency (BCF). Three progression ratios, expressed as percentages, were calculated for the program: Linearity (LIN = VSL/VCL × 100), straightness (STR = VSL/VAP × 100), and wobble (WOB = VAP/VCL × 100). CASA settings adjusted to detect avian spermatozoa (A^2^ = 5 μm^2^). CASA settings for motility were: VCL: 10–100 μm/s; progressive motility >75% STR, circular movement <50% LIN. According to their velocity, the spermatozoa were classified as slow (<10 μm/s), medium (10–50 μm/s) or rapid (>50 μm/s). A minimum of three fields and 500 sperm tracks at a magnification of 100× for each sample were evaluated (image acquisition rate 25 frames/s).

The motility was analyzed as much in fresh samples as freezing–thawed samples of the two experiments. The frozen–thawed samples were diluted in ASG medium [30,31] (64.7 mM Sodium-l-glutamate1H_2_O, 3.1 mM tri-potassium-citrate1H_2_O, 3 mM magnesium acetate4H_2_O, 26.5 mM d-(+)-Glucose monohydrate, 114 mM BES (*N*,*N*-Bis(2-hydroxyethyl)-2-aminoethanesulfonic acid, pH 7.1, osmolality 325 mOsm/kg), 46.2 mM NaOH) with BSA 10 mg/mL before the CASA analysis.

#### 2.5.2. Membrane Integrity

Propidium iodide (PI) and SYBR-14 were used as fluorochromes in the examination of membrane integrity with 200 cells being examined [32]. When conducting this procedure 2 μL of SYBR-14 (1 mM in Me_2_SO diluted 1:19 in Me_2_SO) and 5 μL of the semen sample were added to an Eppendorf tube containing 100 μL of HEPES medium (20 mM Hepes, 197 mM NaCl, 2.5 mM KOH, and 10 mM glucose), and incubated at 5 °C for 10 min in darkness. Afterwards, 1 μL of PI (stock solution: 2.4 mM in water) was added to the mix, and incubated for 2 min at 5 °C in darkness. The samples were then examined using an epifluorescence microscope at 400× (wavelength: 450–490 nm) with fluorescent microscopy (Eclipse E200, Nikon, Japan). Two spermatozoa population were distinguished: Spermatozoa stained green (no PI staining) were considered to be alive, while red colored spermatozoa (PI-positive) and spermatozoa with red and green colors were considered to be dead.

These parameters were analyzed as much in fresh samples as freezing–thawed samples of the two experiments. 

#### 2.5.3. DNA Fragmentation

DNA integrity was measured by terminal deoxynucleotidyl transferase dUTP nick end labelling (TUNEL) as previously described procedures using the kit “In Situ Cell Death Detection” (Roche, Basel, Switzerland) [5]. The samples were diluted in 4% paraformaldehyde, and 10 µL of this dilution were placed on a glass slide and left to dry. Subsequently, the spermatozoa were permeabilized with 0.1% of Triton X-100 in PBS and washed in PBS. After the DNA fragmentation was nick end-labelled with the kit work solution, which content the substrates and the enzyme terminal transferase. The samples were incubated 1 h in humid box at 37 °C. Then the samples were washed in PBS and counterstained with Hoechst at 0.1 mg/mL in PBS for 5 min in the dark. Afterwards, the samples were washed in PBS and mounted using Fluoromount (Sigma-Aldrich, St. Louis, MO, USA). Samples were observed using a fluorescent microscopy (Eclipse E200, Nikon, Japan), and 200 spermatozoa were counted at 400× (wavelength: 510–560 nm).

This parameter was measured only in fresh samples of the second experiment and all freezing–thawed samples of the two experiments.

#### 2.5.4. Mitochondrial and Acrosomal Status

Mitochondrial function and acrosome state were analyzed by Mitotracker Green FM^®^ (MITO, Invitrogen M7514) in the experiment 2, for freezing–thawed samples, as described by Bernal et al., with some modification [33,34]. For this, 5 μL of the semen sample were added to an Eppendorf tube that contains 100 μL of HEPES medium. After, 2 μL of antifade and 0.2 μL of Mitotraker Green were added too. The Eppendorf tubes were incubated 23 min at 5 °C in darkness. The samples were then examined using an epifluorescence microscope at 1000× with fluorescent microscopy (Eclipse E200, Nikon, Japan). Four subpopulations were distinguished: (1) Spermatozoa with intact acrosome and high mitochondrial function, (2) spermatozoa with damaged acrosome and high mitochondrial function, (3) spermatozoa with intact acrosome and low mitochondrial function, and (4) spermatozoa with damaged acrosome and low mitochondrial function.

The percentage of spermatozoa with an intact acrosome was also determined by examining 200 aniline blue-stained cells by phase-contrast microscopy (magnification 1000×), following the procedure of Santiago-Moreno et al. [35]. The dry smears were fixed at room temperature during 30 min in buffered 2% glutaraldehyde in PBS and air-dried. The slides were then stained for 5 min with 5% aqueous aniline blue mixed with 2% acetic acid (pH = 3.5) (Merck, Germany), washed with distilled water and air-dried again [35]. For the aniline blue smears was used a phase-contrast microscope (Zeiss, Oberkochen, Germany) (magnification 1000×) and 200 spermatozoa were analyzed by smear.

#### 2.5.5. Sperm Head Morphometric Analysis

**Figure 1:**
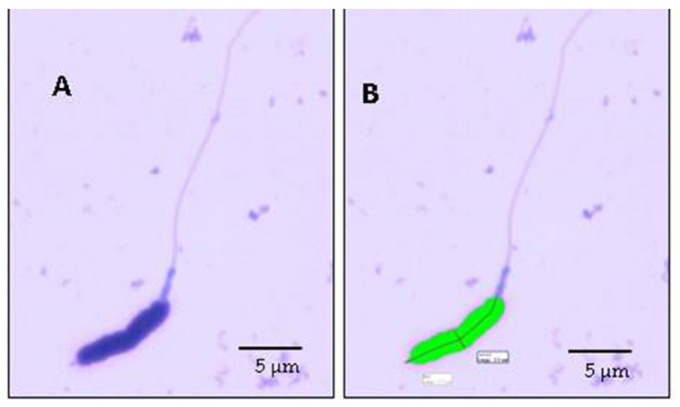
Flamingo sperm cell stained with Hemacolor^®^ (**A**) Image of capture of flamingo sperm, (**B**) image of analyzed capture of flamingo sperm with Motic Image Advance V 3.0 software (Motic Spain, S.L.U., Barcelona, Spain)).

Head morphometry was examined as previously described by Villaverde-Morcillo et al. [36]. Smears were prepared by spreading 5 µL of diluted semen samples onto glass slides and air-dried. Smears were then fixed and stained with Hemacolor^®^ during 2 min each step with corresponding kit’s acid and basic stains, according to the manufacturer´s recommendations [37]. After, all slides were sealed with Eukitt mounting medium (Panreac Quimica S.L.U., Barcelona, Spain) and a coverslip. The Motic Image Advanced V.3.0 software (Motic Spain, S.L.U., Barcelona, Spain) and Motic BA 210 optical microscope (Motic Spain, S.L.U.) were used for the analysis. The captures were done with a Moticam 3+ (Motic Spain S.L.) camera [36]; 25 spermatozoa were captured and their head were measured with acrosome (Figure 1). 

### 2.6. Statistical Analysis

Values for semen characteristics were reported as means ± SE (Standard Error), except the sperm head measurements that were expressed as means ± SD (Standard Deviations). For several variables, there was not a normal distribution when there was assessment using the Shapiro–Wilk’s test. The Mann–Whitney test was used to compare the effect within frozen–thawed samples between the two cryoprotectant agents, and the Wilcoxon test for matched pairs was used to compare the effect within frozen–thawed samples with presence or absence of seminal plasma. The Mann–Whitney test was used to compare the percentage of sperm with intact acrosome according aniline blue assay. To assess the response to freezing-thawing in both experiments, a cryoresistance ratio [38,39] was determined for each of the semen variables as follows:

*Cryoresistance ratio* (*CR*) = (*value after thawing* (*post*)/*value before thawing* (*pre*)) × 100.

Differences in the CRs for the first experiment were compared using the Mann–Whitney test and for the second experiment were compared using the Wilcoxon test. All calculations were performed using Statistica software v.13 (Dell Statistica, StatSoft Inc., Tulsa, OK, USA).

## 3. Results

The sperm head measurements (mean ± SD) reported by computerized morphometric analysis were: 11.3 ± 1.2 µm length, 2.3 ± 0.5 µm width, 27.4 ± 3.1 µm perimeter, and 21.6 ± 4.6 µm^2^ area. Sperm characteristics of fresh samples are shown in Table 1 and Table 2.

### 3.1. Experiment 1: Effect of DMA 6% and Me_2_SO 8% in the Cryopreservation

The mean semen volume was 20.9 ± 5.1 µL, and the mean sperm concentration was 1228.2 × 10^6^ ± 348 × 10^6^ spermatozoa/mL. The total number of sperm per ejaculate was 27.1 × 10^6^ ± 11.6 × 10^6^ spermatozoa.

There were no significant differences for any sperm variable between samples frozen with 6% DMA and those frozen in 8% Me_2_SO (Table 1). There were also no differences in any of the values for the cryoresistance ratios (Table 3).

### 3.2. Experiment 2: Effect of Seminal Plasma in the Cryopreservation

The mean semen volume was 40.3 ± 6.6 µL, and the mean sperm concentration was 1912.2 × 10^6^ ± 713.9 × 10^6^ spermatozoa/mL. The total number of sperm per ejaculate was 43.3 × 10^6^ ± 10.7 × 10^6^ spermatozoa. Significant differences were found for the parameters VSL, WOB, intact acrosome percentage (aniline blue-stained) (*p* < 0.05), LIN and STR (*p* < 0.01). These parameters were higher in samples frozen with seminal plasma than samples frozen without seminal plasma, except the percentage of spermatozoa with intact acrosome (Table 2). 

Different letters indicate significant differences (lower case *p* < 0.05; capital letter *p* < 0.01) within each sperm variable, between samples with or without seminal plasma. Mitotraker (MITO), Acrosome (ACRO), Curvilinear velocity (VCL), straight line velocity (VSL), average path velocity (VAP), linearity (LIN), straightness (STR), wobble (WOB), amplitude of lateral head (ALH), beat-cross frequency (BCF). Results are expressed as mean ± SE.

CR values were higher for samples in presence of seminal plasma that samples in absence of seminal plasma for the following parameters: Progressive motility, VCL, VSL, VAP, WOB (*p* < 0.05), LIN, and STR (*p* < 0.01) (Table 4).

## 4. Discussion

This study is the first describing the sperm characteristics and sperm head morphometric variables in flamingos. The length of head (11.3 µm) is similar to turkey and emu, but the width (2.3 µm) is similar to gyrfalcon, which makes its area largest (21.6 µm^2^) [40]. Head morphometric data provide relevant information in future studies about sperm cryobiology in these species because the head size is related with the ability of sperm to survive the freeze/thawed process [40]. 

There were no apparent differences among cryoprotectants regarding its effects on frozen–thawed sperm variables. However, the wide individual differences could explain the lack of significance for many variables. The removal of the seminal plasma provided lower results after thawing than in samples with seminal plasma.

DMA and Me_2_SO are permeating cryoprotectant which act intra- and extracellularly inducing spermatozoa dehydration [41], osmotic stress [42], and even membrane fusion at high concentrations [43]. Despite this, the use of these cryoprotectants at concentrations of 6% of DMA and 8% of Me_2_SO resulted in high post-thaw plasma membrane integrity, viability, acrosomal integrity, motility and fertility in sperm from chicken, crane or Indian red jungle fowl [3,10,13]. The findings in the present study did not reveal differences between the two cryoprotectants. This fact may be related to the similar cryoprotective mechanism of both cryoprotectants. They share physical-chemical properties as two hydrophobic methyl groups that can create three hydrogen bonds with water [44]. Even though there were no differences, we decided to use Me_2_SO in the experiment 2 because the toxic effect of DMA when high concentrations are used [29]. Our findings showed poor motility values after thawing, which may be explained in part by the low motility values in fresh (10% progressive motility). Despite the low initial motility, the reduction of progressive motility after freezing-thawing process was 50%. These values agree with previous freeze–thawing procedures in chicken rooster sperm with fertilization capacity [6].

The seminal plasma is a complex mixture of proteins, free amino acids, lipids, carbohydrates, and hormones [5,20,21,22,23,24]. In the second experiment, it was decided to remove the seminal plasma because other studies reported a deleterious effect in the storage of semen samples of chicken and turkey [25,26,27]. Our findings revealed better motility results in presence of seminal plasma. This may be due to the presence of certain proteins in the seminal plasma that might contribute to the cryoprotectant permeability [45]. Moreover, free amino acids content of flamingo’s seminal plasma might improve sperm kinetic activity; for instance, glutamine and proline improve sperm motility variables in goat sperm [5,46]. Also, it has been shown that certain amino acids might stabilize the membranes when interacting with the lipid bilayer [47], protect spermatozoa against toxic solutes [48], and induce cell dehydration during freezing process [49]. Blesbois and Reviers’s studies [50] showed that the high molecular weight fractions of seminal plasma appeared to enhance fertilizing ability in fowl, which might be related to best values of motility as we see in this research. Some seminal plasma components, as reduced glutathione (GSH), glutathione peroxidase (GPx), phospholipid hydroperoxide glutathione peroxidase (PHGPx), and superoxide dismutase (SOD) [51,52] might contribute to reduce the concentration of reactive oxygen species, and thus improve the sperm motility [53,54]. 

Other hypothesis is the seminal plasma action on spermatozoa through exosomes, as it has been described in mammals. These exosomes are membranes vesicles of 30–120 nm coming from organs of male genital tract that may regulate some physiologic process of the spermatozoa as capacitation, acrosome reaction and anti-oxidation [55,56]. Some works have described that the human, canine, equine and bovine prostasomes are able to produce extracellular ATP thanks of content glycolytic enzymes of their inside [57,58]. Some studies have shown that extracellular ATP improves sperm motility [59,60,61]. Extracellular ATP might be used by some membrane calcium ATPase, which presence has been described in murine sperm, and this might produce a calcium flow in the spermatozoa [62]. In addition, the prostasomes can induce a calcium flow through the transfer of progesterone receptors, cyclic adenosine diphosphoribose (cADPR)-synthesizing enzymes, ryanodine receptors (RyRs), and other Ca^2+^ signaling tools by their fusion with the sperm [56]. Calcium flow improves the motility variables and induces acrosome reaction [63]. This may explain our findings related to a higher VSL, WOB, LIN, and STR but lower acrosome integrity in frozen/thawed samples in presence of seminal plasma.

The low percentage of motile sperm determines that a high number of sperm should be used in each dose for artificial insemination (AI). Taking into account the sperm quality after freezing-thawing (about 45% viability and 15 % motility) along with the criteria for AI in wild poultry [10], we estimate that at least two consecutive intravaginal AI procedures (3 days apart) involving 400 million sperm (two straws)/female at each insemination should be required for a successful fertility. More effective overall cryopreservation protocols (e.g., optimizing cooling rates and researching new additives) and eventually fertility trials using intravaginal insemination techniques should be approached in future studies.

## 5. Conclusions

This is the first report describing sperm characteristics and cryopreservation procedures in a flamingo species. Our findings revealed that the use of DMA 6% or Me_2_SO 8% did not show differences; we recommend the use of Me_2_SO because the cell toxic effect of DMA. On the other hand, the removal of seminal plasma did not contribute a benefit in the cryopreservation method. 

## Figures and Tables

**Table 1:** American flamingo sperm variables in fresh and froze-thawed samples using two cryoprotectant agents (8% Me_2_SO and 6% DMA).

Sperm Variables	8% Me_2_SO		6% DMA	
	Fresh	Thawed	Fresh	Thawed
Motility (%)	37.5 ± 9.9	10.0 ± 3.1	42.5 ± 9.0	17.0 ± 7.0
Score	2.2 ± 0.6	1.7 ± 0.3	2.5 ± 0.5	1.4 ± 0.4
Viability (%)	78.6 ± 2.3	43.6 ± 3.5	80.1 ± 3.1	48.2 ± 3.8
DNA damage (Tunel + %)	-	17.2 ± 3.5	-	16.5 ± 5.7
Static (%)	67.6 ± 9.2	82.7 ± 4.2	61.2 ± 9.2	85.1 ± 5.2
No progressive motility (%)	23.2 ± 6.2	12.7 ± 2.8	27.7 ± 6.3	9.5 ± 3.2
Progressive motility (%)	9.2 ± 3.9	4.6 ± 1.5	11.1 ± 3.9	5.4 ± 3.4
VCL (µm/s)	36.3 ± 8.0	33.1 ± 5.9	40.0 ± 7.7	29.4 ± 11.2
VSL (µm/s)	22.5 ± 6.4	22.1 ± 4.6	26.1 ± 6.1	22.1 ± 10.0
VAP (µm/s)	28.2 ±7.3	25.9 ± 5.0	31.8 ± 6.9	24.8 ± 10.4
LIN (%)	47.8 ± 8.0	58.7 ± 7.5	53.4 ± 7.7	48.5 ± 11.0
STR (%)	64.7 ± 8.6	75.7 ± 8.7	69.6 ± 8.5	61.1 ± 12.2
WOB (%)	64.0 ± 8.4	69.2 ± 8.1	67.7 ± 8.3	59.2 ± 11.3
ALH (µm)	1.8 ± 0.4	1.6 ± 0.3	2.0 ± 0.4	1.1 ± 0.4
BCF (Hz)	5.9 ± 1.3	6.3 ± 1.2	6.9 ± 1.2	4.5 ± 1.5
Acrosome integrity (%)	91.8 ± 2.0	73.9 ± 3.6	91.8 ± 2.0	63.0 ± 3.0

Curvilinear velocity (VCL), straight line velocity (VSL), average path velocity (VAP), linearity (LIN), straightness (STR), wobble (WOB), amplitude of lateral head (ALH), beat-cross frequency (BCF). Results are expressed as mean ± SE.

**Table 2:** American flamingo sperm variables in fresh and frozen–thawed samples with or without seminal plasma.

Semen Variables	Fresh	Thawed	
		With Seminal Plasma	Without Seminal Plasma
Motility (%)	51.4 ± 7.5	11.4 ± 3.4	9.0 ± 3.1
Score	2.2 ± 0.2	2.2 ± 0.2	1.9 ± 0.3
Viability (%)	78.0 ± 4.5	44.0 ± 5.0	45.4 ± 2.8
DNA damage (Tunel + %)	12.1 ± 3.4	32.9 ± 3.8	29.1 ± 4.0
Mito+, Acro+ (%)	-	29.6 ± 4.1	35.9 ± 4.3
Mito+, Acro− (%)	-	49.5 ± 3.8	45.9 ± 4.5
Mito−, Acro+ (%)	-	4.3 ± 1.4	3.9 ± 1.9
Mito−, Acro− (%)	-	16.6 ± 2.3	14.3 ± 2.8
Static (%)	56.5 ± 8.3	87.6 ± 2.0	88.9 ± 2.8
No progressive motility (%)	34.5 ± 6.3	9.3 ± 1.4	9.0 ± 2.1
Progressive motility (%)	9.0 ± 3.9	3.1 ± 0.7	2.1 ± 0.8
VCL (µm/s)	39.3 ± 5.7	35.0 ± 3.0	29.5 ± 4.1
VSL (µm/s)	20.1 ± 4.0	22.4 ± 2.5 ^a^	16.6 ± 3.3 ^b^
VAP (µm/s)	28.2 ± 5.0	26.9 ± 2.7	21.6 ± 3.8
LIN (%)	47.0 ± 3.6	62.7 ± 2.7 ^A^	52.0 ± 4.4 ^B^
STR (%)	67.6 ± 3.1	82.4 ± 1.6 ^A^	73.8 ± 3.2 ^B^
WOB (%)	68.4 ± 2.7	75.8 ± 1.9 ^a^	69.4 ± 3.6 ^b^
ALH (µm)	2.5 ± 0.3	1.5 ± 0.3	1.3 ± 0.3
BCF (Hz)	6.5 ± 1.0	6.0 ± 1.1	5.1 ± 1.3
Acrosome integrity (%)	-	55.6 ± 2.7 ^b^	66.9 ± 3.8 ^a^

Different letters indicate significant differences (lower case *p* < 0.05; capital letter *p* < 0.01) within each sperm variable, between samples with or without seminal plasma. Curvilinear velocity (VCL), straight line velocity (VSL), average path velocity (VAP), linearity (LIN), straightness (STR), wobble (WOB), amplitude of lateral head (ALH), beat-cross frequency (BCF). Results are expressed as mean ± SE.

**Table 3:** Cryoresistance ratio of American flamingo sperm variables frozen with two cryoprotectant agents (8% Me_2_SO and 6% DMA).

Cryoresistance Ratio	8% Me_2_SO	6% DMA
Motility	17.0 ± 6.1	40.2 ± 19.1
Score	49.7 ± 13.7	51.5 ± 19.2
Viability	56.3 ± 5.0	56.2 ± 8.0
Static	153.3 ± 31.7	183.8 ± 40.3
No progressive motility	71.3 ± 25.4	94.5 ± 55.7
Progressive motility	70.8 ± 27.7	122.5 ± 72.6
VCL	70.6 ± 16.5	106.5 ± 44.1
VSL	96.3 ± 28.1	170.6 ± 73.5
VAP	80.1 ± 21.0	129.4 ± 52.8
LIN	102.9 ± 22.2	89.0 ± 24.8
STR	92.1 ± 16.8	76.2 ± 19.6
WOB	86.8 ± 15.5	75.0 ± 18.5
ALH	48.5 ± 12.8	32.4 ± 13.5
BCF	63.7 ± 16.6	44.9 ± 18.5
Acrosome integrity	82.2 ± 6.0	70.3 ± 1.5

Curvilinear velocity (VCL), straight line velocity (VSL), average path velocity (VAP), linearity (LIN), straightness (STR), wobble (WOB), amplitude of lateral head (ALH), beat-cross frequency (BCF). Results are expressed as mean ± SE.

**Table 4:** Cryoresistance ratio of American flamingo sperm variables frozen with or without seminal plasma.

Cryoresistance Ratio	With Seminal Plasma	Without Seminal Plasma
Motility	27.4 ± 9.0	26.3 ± 13.2
Score	112.7 ± 19.9	85.3 ± 9.6
Viability	56.1 ± 6.0	60.6 ± 5.7
Tunel+	472.7 ± 166.8	599.7 ± 265.9
Static	244.4 ± 84.3	241.6 ± 78.4
No progressive motility	46.9 ± 12.8	51.1 ± 21.5
Progressive motility	138.7 ± 87.6 ^a^	54.9 ± 30.7 ^b^
VCL	106.0 ± 18.0 ^a^	89.3 ± 20.4 ^b^
VSL	152.8 ± 32.2 ^a^	112.6 ± 37.7 ^b^
VAP	122.5 ± 25.7 ^a^	98.1 ± 30.0 ^b^
LIN	139.4 ± 8.2 ^A^	113.2 ± 8.2 ^B^
STR	124.0 ± 4.8 ^A^	110.0 ± 3.5 ^B^
WOB	111.7 ± 3.1 ^a^	102.0 ± 5.1 ^b^
ALH	74.2 ± 28.6	64.3 ± 27.7
BCF	103.2 ± 27.6	87.5 ± 32.2

Different letters indicate significant differences (lower case *p* < 0.05; capital letter *p* < 0.01) within each sperm variable, between samples with or without seminal plasma. Curvilinear velocity (VCL), straight line velocity (VSL), average path velocity (VAP), linearity (LIN), straightness (STR), wobble (WOB), amplitude of lateral head (ALH), beat-cross frequency (BCF). Results are expressed as mean ± SE.

## Data Availability

The data presented in this study are available on request from the corresponding author.
